# Life-history theory in psychology and evolutionary biology: one research programme or two?

**DOI:** 10.1098/rstb.2019.0490

**Published:** 2020-06-01

**Authors:** Daniel Nettle, Willem E. Frankenhuis

**Affiliations:** 1 Population Health Sciences Institute, Newcastle University, Newcastle upon Tyne NE1 7RU, UK; 2 Behavioural Sciences Institute, Radboud University, Nijmegen 6500 HE, Netherlands

**Keywords:** life-history theory, psychology, review, research programmes

## Abstract

The term ‘life-history theory’ (LHT) is increasingly often invoked in psychology, as a framework for integrating understanding of psychological traits into a broader evolutionary context. Although LHT as presented in psychology papers (LHT-P) is typically described as a straightforward extension of the theoretical principles from evolutionary biology that bear the same name (LHT-E), the two bodies of work are not well integrated. Here, through a close reading of recent papers, we argue that LHT-E and LHT-P are different research programmes in the Lakatosian sense. The core of LHT-E is built around ultimate evolutionary explanation, via explicit mathematical modelling, of how selection can drive divergent evolution of populations or species living under different demographies or ecologies. The core of LHT-P concerns measurement of covariation, across individuals, of multiple psychological traits; the proximate goals these serve; and their relation to childhood experience. Some of the links between LHT-E and LHT-P are false friends. For example, elements that are marginal in LHT-E are core commitments of LHT-P, and where explanatory principles are transferred from one to the other, nuance can be lost in transmission. The methodological rules for what grounds a prediction in theory are different in the two cases. Though there are major differences between LHT-E and LHT-P at present, there is much potential for greater integration in the future, through both theoretical modelling and further empirical research.

This article is part of the theme issue ‘Life history and learning: how childhood, caregiving and old age shape cognition and culture in humans and other animals’.

## 1. Introduction

**Figure 1.**
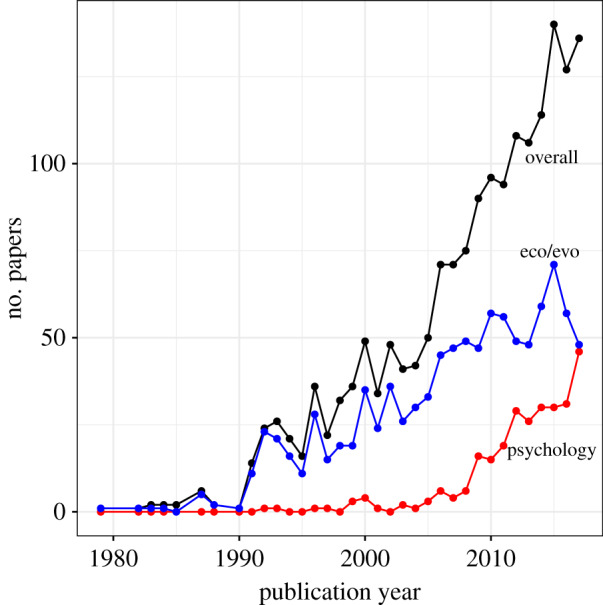
Number of papers per year using the term ‘life-history theory’ in title, abstract or keywords overall; in journals whose subject category includes ecology, evolutionary biology or zoology; and in journals whose subject category includes psychology. Results are from a Web of Science search (www.webofscience.com) for complete years up to and including 2017. Note that theoretical work on life history in evolutionary biology goes back further than implied by these data (see §3). Earlier authors preferred the term ‘life-history evolution’. (Online version in colour.)

This special issue brings together research from psychology, and learning in particular, with research on life history, which is more typically concerned with growth, physical maturation and senescence. The desire to integrate psychology with life history is not a new one. It has been going on for some time under the banner of ‘life-history theory’ (LHT). LHT originated in evolutionary biology, but in the last 15 years, the term has appeared more and more in the psychology literature, particularly in personality psychology and parts of developmental psychology. Indeed, if present trends continue, it will soon be as frequently encountered in psychology as it is in evolutionary biology (figure 1).

We have recently shown quantitatively that papers from psychology which invoke LHT don't tend to cite many of the same references as papers from evolutionary biology (under which term we also included ecology) that invoke LHT [1]. This has become particularly true in the period since 2010. Prior to that date, most papers invoking the LHT terminology drew on the same core set of key theoretical references, regardless of their discipline. After 2010, LHT papers from psychology began to draw on a set of core theoretical references of their own, with little direct citation to works from evolutionary biology. These findings raise the question of whether the ‘theory’ in ‘LHT’ is actually the same one in the two cases. In working on our quantitative review, it struck us how different the presentation of the basic principles of the theory was in the papers from psychology as compared to those from evolutionary biology. We concluded the literature suffers from the so-called ‘jingle fallacy’: the sometimes false expectation that if two things bear the same name, they must be equivalent. In this paper, we aim to qualitatively document these differences, give a brief historical analysis of how they arose, and make suggestions for how to move forward. To be clear from the outset, we do not aim to *evaluate* either the claims or the methods of LHT in psychology or in evolutionary biology. Different aims generate different traditions of theorizing, different methods and different results. These cannot necessarily be judged ‘better’ or ‘worse’ than one another. Our aim is merely to compare what the term LHT currently means in the two disciplines, and hence discuss possible future directions.

We base our analysis around the concept of research programmes [2]. The central question is whether life-history theory in psychology (henceforth LHT-P) and life-history theory in evolutionary biology (LHT-E) constitute the same research programme or not. Authors in LHT-P typically claim that they do. For example, one finds sentences such as ‘LHT, a branch of evolutionary biology, has demonstrated that the human brain is designed to respond adaptively to variations in resources in the local environment’ [3, p. 2]. It is LHT-E that is a branch of evolutionary biology, but primarily LHT-P that concerns the human brain and its responses to the local environment. Thus, the sentence conflates the two. Whether it is valid to do so is in part a ‘ship of Theseus’ problem. The ship of Theseus problem asks, if every plank of a wooden ship is successively replaced, under what circumstances is it appropriate to speak of it still being the same ship? In the present instance, the puzzle is: if a branch of evolutionary biology is extended to new kinds of phenomena and methods, under what circumstances is it still a branch of evolutionary biology?

Research programmes in science have a number of typical characteristics. First, they have a *hard core* of assumptions and principles: this consists of the ‘assumptions so basic that to question their validity would be tantamount to abandoning [the programme]’ [4, p. 6]. Second, they have suites of *auxiliary hypotheses*: these are ideas that arose from the programme, but could be superseded or rejected without putting the overall programme in jeopardy. Third, they have *methodological rules*. These dictate what are viewed as good grounds for proposing hypotheses or counting claims as having been supported, or refuted. Finally, research programmes often contain *analogical extensions* of their theories [5]. For example, the idea that variation and selection may be used to explain change over time in technologies or cultural traditions [6] is an analogical extension of Darwinian evolutionary theory. The failure of an analogical extension would not lead to the failure of the parent research programme. However, it is sometimes difficult to say where the parent research programme ends and an analogical extension has begun. Whether LHT-P is usefully viewed as the same research programme as LHT-E depends on whether one sees the extensions made by LHT-P as direct application of theories, or just analogies; whether the modifications that have been made are to the hard core or just to auxiliary hypotheses of LHT-E; and whether the same methodological rules still apply in the two cases.

In §2, we give examples of how the principles of LHT are typically presented in ecological and in psychological papers, based on our systematic search [1] and informal reading of the literature. This leads us to generalizations about what appear to be the core tenets of the research programme in the two cases. The next two sections trace the histories of LHT-E (§3) and LHT-P (§4), in an attempt to understand how the core tenets have become so different. Section 5 attempts to synthesize: we argue that LHT-E and LHT-P share many historical links, but are, as it stands, quite different research programmes. However, they could become more closely linked in the future. We should add a caveat about our methodology: our characterizations of LHT-E and LHT-P are based on randomly selected papers using the term LHT. Our search strategy makes no distinction between the most nuanced, most accurate, most rigorous presentations of the theory, and the loosest. Thus, our characterization is a rough picture of what LHT *typically* means when the term is used in psychology or in evolutionary biology, not a detailed review of the best or most nuanced presentations.

## 2. Presentation of ‘life-history theory’ in evolutionary and in psychological papers

In papers from evolutionary biology, the prototypical claims ascribed to LHT are: (i) there are trade-offs between different components of fitness (e.g. survival and reproduction, quality and quantity of offspring) that prevent their simultaneous maximization; (ii) natural selection acts on life-history traits, leading to trait-values that maximize fitness; and (iii) (therefore) populations inhabiting different ecologies or demographies will evolve different patterns of life-history trait values. Example quotations are shown in table 1. Note these claims are all fairly general. Researchers are typically interested in testing a more specific prediction than any of (i)–(iii). However, those specific predictions are not usually presented as properties of LHT *per se*. Rather, they arise from lower-level models or hypotheses that were generated by LHT, but are not constitutive of it. A few exceptions—where a very specific claim is described as being ‘predicted by LHT’, rather than by a specific model or sub-theory—are shown in the final row of table 1.

**Table 1.** Typical tenets of life-history theory as presented in the introductions of papers from evolutionary biology.

tenet	description	examples
(i)	trade-offs exist	‘LHT assumes that reproduction and lifespan are constrained by trade-offs which prevent their simultaneous increase’ [7, p. 483] ‘LHT predicts a tradeoff between reproduction and survival’ [8, p. 719] ‘LHT predicts a trade-off between current and future reproduction for iteroparous organisms’ [9, p. 1323] ‘LHT predicts a trade-off between offspring quality and quantity’ [10, p. 7780]
(ii)	selection acts on life-history traits, leading to fitness maximization	‘LHT predicts that evolutionary forces should shape the timing of life events such as development, maturation, reproduction and death’ [11, p. 408] ‘LHT posits that organisms allocate energy….to primary life tasks…in a manner that maximizes fitness’ [12, p. E3914] ‘LHT predicts that organisms optimize their resource allocation strategy to maximize lifetime reproductive success’ [13, p. 347] ‘the….tradeoffs between costly processes such as reproduction and self-maintenance are predicted to be resolved such that the conversion of resources into fitness is maximised’ [8, p. 720]
(iii)	different ecologies and demographies produce different life histories	‘LHT predicts that populations experiencing different patterns of age- or size-specific mortality will evolve divergent life histories’ [14, p. 249]
(iv)	specific predictions	‘LHT predicts a single optimal offspring size’ [15, p. 168] ‘LHT suggests that growth is biphasic for many organisms, with a change-point in growth occurring at maturity’ [16, p. 182] ‘LHT ….predicts delayed development when non-density dependent mortality is low…’ [17, p. 1]

Tenet (i) of table 1 has implications for the study of individuals; under tenet (i), other things being equal, an individual allocating more energy to reproduction must have less energy to allocate to survival. Tenets (ii) and (iii), however, are best interpreted as concerning population averages and population processes. Tenet (ii) says, at most, that the average individual from a population will show a pattern of life-history traits that maximizes fitness for the statistical composite of environments its ancestors experienced. Individuals will be scattered around the population average for any particular trait—for example because of genetic mutation and recombination—and tenet (ii) does not require that LHT make any particular adaptive claim about this scatter. Tenet (iii) of table 1 entails a claim that differences between populations or species in terms of average life-history traits might reflect evolutionary adaptation to the ancestral environment; but not necessarily that differences between individuals within the same population are adaptations to the personal environment (that is, the environment experienced by that specific individual in its lifetime). Neither tenets (ii) or (iii) necessarily entail that individuals have any plasticity to shift their life-history trait values according to the personal environment. This plasticity claim is sometimes made in LHT-E (e.g. in [18]), and there are evolutionary life-history models incorporating plasticity [19]. However, claims about plasticity are not ubiquitous within LHT-E.

In summary, the hard core of LHT-E explicitly consists of the idea that there are evolutionary trade-offs; that natural selection acts on life-history traits; and that as a result of this, populations or species experiencing different ecologies and demographies end up with divergent patterns of life-history traits. Although there are many more specific claims and predictions, those are, for the most part, seen as auxiliary rather than hard core: they have arisen from specific models generated by LHT, models that might be superseded or refined. There is also another important principle that is clearly inherent in LHT-E: a statement counts as a ‘prediction’ within LHT-E if there is a formal model showing that statement maximizes fitness under some set of assumptions. This methodological rule makes sense because LHT-E aims to provide ultimate-level explanations of phenotypes that result from interacting selective forces [20].

Papers from psychology often have the same starting point as those from evolutionary biology: the existence of trade-offs (see table 2 for examples). The tenets thereafter, however, tend to be different. Psychology papers almost universally allude to idea that multiple traits covary along a principal axis known as the fast–slow continuum (tenet (ii) of table 2). In our quantitative review [1], we found the fast–slow continuum alluded to in 20 out of 20 recent psychological papers we sampled, and just 2 out of 20 papers from non-human research (in one of those, it only appeared in the Discussion). In psychology papers, the suite of traits related to the fast–slow continuum invariably includes not only classical life-history traits such as timing of maturation or reproduction, but also psychological variables such as attitudes to risk, ability to delay gratification, religiosity, prosociality, optimism and others.

**Table 2.** Typical tenets of life-history theory as presented in the introductions of papers from psychology.

tenet	description	example
(i)	trade-offs exist	‘according to LHT, the finite nature of resources available to organisms during evolution induced multiple-trait trade-offs among fitness components such as current versus future reproduction and offspring quality versus quantity’ [21, p. 1] ‘LHT posits that organisms face important trade-offs in how they allocate….resources among the several competing demands of life….’ [22, p. 889]
(ii)	life-history traits covary between individuals along a fast–slow continuum	‘LHT suggests that humans fall along a spectrum from early reproduction and allocation of resources toward mating effort, to later reproduction and devotion of resources toward somatic and parental effort…referred to as the fast–to–slow life history continuum’ [23, p. 933] ‘Life history strategies vary along a fast/slow continuum’ [24, p. 23] ‘From the perspective of LHT, a mid-level evolutionary framework, [behavioural] phenotypic variables are conceptualized as indicators of individual differences along a fast–slow LH continuum’ [25, p. 1]
(iii)	people adapt to their personal environments, especially those of childhood, by becoming ‘faster’ or ‘slower’.	‘LHT predicts that people calibrate their reproductive strategies to local levels of environmental harshness and unpredictability…’ [26, p. 434] ‘according to LHT….the nature of an individual's childhood environment disposes that individual to adopt a *fast* or a *slow* life history strategy….’ [27, p. 621] ‘these ‘fast’ versus ‘slow’ life history trajectories are strategic responses to the particular environment in which people find themselves’ [28, p. 891] ‘according to LHT, exposure to harshness and/or unpredictability early in life should promote a fast life history strategy’ [29, p. 1542]
(iv)	specific predictions	‘the evolutionary framework of LHT predicts that preferences for risk and delay in gratification should be influenced by mortality and resource scarcity’ [30, p. 1015] ‘LHT predicts that an array of [crime-related] behaviors will shift in response to life expectancy cues’ [31, p. 12] ‘LHT suggests that adult reward sensitivity should be best explained by childhood, but not current, socioeconomic conditions’ [32, p. 48] ‘the evolutionary framework of LHT predicts that preference for delay of gratification should be influenced by social economic status’ [33, p. 1]

A third recurring tenet is that *individuals* strategically adjust their (broadly defined) life-history trait values according to their personal environments (table 2, third row), making LHT-P a theory of individual differences, and especially of individual differences attributed to phenotypic plasticity. Early childhood experience is often viewed as an essential input, though genotypic variation may be acknowledged, too. Note the shift from average trait-values of populations being gradually shifted by selection over evolutionary time (LHT-E), to individuals shifting their phenotypes in response to their personal environments over the course of development (LHT-P). Finally (tenet (iv) of table 2), in LHT-P, many very specific predictions are described as issuing directly from LHT itself, rather than from more specific models or sub-theories. These predictions are quite variable, both in the outcome traits they concern, and what the theory is stated to predict. For example, some studies test for effects of acute psychological manipulations in adulthood on putatively ‘fast’ psychological variables, claiming that LHT predicts an immediate response [33]; whereas others take LHT to specifically predict that childhood experience, rather than the adult context, will have set the values of these traits [32]; and still others take LHT to predict non-additive interactions between childhood experience and adult context in determining the trait value [30].

In our view (and others may disagree), the differences between LHT-E and LHT-P that we have outlined above are more than additional auxiliary hypotheses that LHT-P has added to the core of LHT-E. Instead, the hard cores of the programmes, as described by the papers themselves, appear to be different. LHT-P focuses on individual differences, along a fast–slow continuum, mainly as a result of phenotypic plasticity. These commitments *constitute* the research programme. The following quotations, with emphasis added by us, illustrate this point:

LHT provides an evolutionary *account of individual differences in various traits*, including wellbeing. *The theory distinguishes* between a fast LH strategy, indicated by a short-term perspective (e.g., impulsivity), versus a slow LH strategy [34, p. 277]

LHT is an evolutionary framework that explains individual differences [29, p. 1543]

we draw on LHT, *which concerns* the relationship between childhood poverty and adulthood preferences for security [24, p. 21]

*at its core*, LHT is a *motivational* framework, whereby motivational ‘states’ are determined by the specific problems and opportunities associated with an organism's current develop-mental stage and local ecology [35, p. 1]

By contrast, it would be perfectly possible, and indeed normal, to be interested primarily in interspecific differences, to make no reference to the fast–slow continuum, to plasticity or to proximate motivation, and yet still describe one's work as LHT-E. Thus, our reading of the literature leads us to conclude that LHT-E and LHT-P are largely different research programmes. They differ not just in some extra species-specific auxiliary hypotheses or empirical methods, but rather in their core tenets. In §§3 and 4 we trace historically how this situation has come to pass, before turning, in §5, to suggestions for the future.

## 3. Life-history theory in evolutionary biology

Life-history traits are those that figure directly in reproduction and survival, such as size at birth, age at maturity, number and timing of offspring, and age at senescence. Biologists had long appreciated that individuals of different species differ dramatically in the value of such traits. From the 1950s onwards, theorists began to explore mathematically how variation in life-history traits would affect fitness, and hence how the values of those traits might be shaped by natural selection [36–41].

An important early proposal was the idea that species could be characterized as more *r­-*selected or more *K*-selected. This idea derived from modelling work by MacArthur & Wilson [42]. The former have been selected to maximize their population growth rates when population density is low, while the latter have been selected to maximize their survival at high population densities (*r* and *K* refer to corresponding terms in the logistic population growth equation). Influentially, Pianka [43] proposed a suite of traits that ought to go with *r* and *K* (body size: small for *r*, larger for *K*; age at maturation: early for *r*, later for *K*; fecundity: high for *r*, lower for *K*, etc.). The *r*/*K* framework had two roles. The first was descriptive generalization: that species might be arrayed on a single continuum with fast reproduction and its correlates at one end and slow reproduction at the other. The second was an evolutionary explanation of the descriptive generalization: owing to the environments they live in, *r*-selected species have been more shaped by selection for a high maximal population growth rate, and *K*-selected species have been more shaped by selection to thrive under competition when populations are dense. The suite of traits proposed by Pianka to go along with *r* and *K* respectively were not mentioned in MacArthur and Wilson's original work [42] and did not arise from any formal model: they were illustrative suggestions, originally developed for Pianka's undergraduate population biology class [44].

Historians of LHT-E agree that the *r/K* framework was influential and attracted people to the field, but was eventually largely abandoned [44,45]. The descriptive part was seen as too simplistic. Debates about how much inter-species variation can be explained with a principal axis go on to this day [46–50]. Though life-history traits do tend to covary across species, the strength of a principal-axis pattern depends on the level of entity sampled (populations, species, or higher taxa); how phylogenetic relatedness is handled; whether body size, which scales allometrically with many other traits, is corrected for; the statistical methods used; and which traits are included. Regardless of the outcome of these empirical matters, though, the evolutionary explanation part of the *r/K* framework was also abandoned. The different modes of selection supposed to underlie *r* and *K* were never demonstrated, and artificial selection experiments did not support the predictions [44] (though interest in the different effects of density-dependent and density-independent selection on life-history traits continues to this day [51]).

With the decline in interest in the *r/K* framework, LHT-E began to focus on models built around other factors, such as age-specific patterns of mortality. These ‘demographic’ models (summarized in [45,52]) went on to be synonymous with the term LHT. These models predict that a wide variety of patterns can be produced by selection under different environmental and demographic regimes, and organismal constraints. Hence the conclusion that:

There are virtually no general predictions in life history theory because some organism can always be found with a tricky and unexpected trade-off…Thus, it is more sensible to treat the theory as a general framework that tells us what questions need to be answered when building a model for some particular organism, than it is to try to use the predictions of general models [45, p. 208]

The consequence is that, rather than being able to collect a ready-made LHT prediction off the shelf for some new trait (e.g. a psychological one) in some new organism (e.g. humans), we would have to build a model fit for that purpose instead:

If you are interested in testing LHT, then collaborate with a theoretician and build a model of your particular organism, testing both assumptions and predictions against your data. There are few predictions…general enough to be convincingly and fairly tested on some randomly chosen organism without modification [45, p. 208]

In light of these comments, researchers in evolutionary biology are left with mostly general claims to constitute the hard core of the programme (there are trade-offs, and life-history traits are under selection). More detailed predictions are often model-specific and auxiliary to the programme. It would be wrong to argue that there are no mid-level generalizations in between (see the examples under tenet (iv) of table 1), but these are perhaps fewer and less clear-cut than is commonly assumed in psychology. However, a clear and defining asset of LHT-E is its methodological rules: the mathematical modelling techniques exist, and are generally agreed upon. Thus, to do LHT-E is to build explicit mathematical models; to do so in particular ways; and to test model assumptions and predictions for empirical cases. It is these activities more than any specific set of predictions about any particular species that constitute the research programme.

## 4. Life-history theory in psychology

Early applications of ideas from LHT-E in psychology explicitly referenced the *r/K* framework. Indeed, some explicitly described themselves as ‘differential K’ theory [53]. That is, humans are generally *K*-selected, but some humans are more so than others. Thus, the *r/K* contrasts suggested by Pianka [43] to hold at the species level were being extended to capture differences between conspecifics. These works picked up on two features from Pianka [43]: the first was that while early age at first reproduction was the master signature of being more *r-*selected, a whole suite of other traits might be needed to support it. For the psychologists, these included behaviours or cognitive traits, although these had not featured in Pianka's account. The second was that *r*-selection dominated particularly when the environment was variable and/or unpredictable. For Pianka [43] this referred to variability and unpredictability over evolutionary timescales. Some early work in LHT-P retained this focus and argued, controversially, that different human populations had experienced different selective histories [53]. However, the focus soon shifted from differences between populations and experience over evolutionary timescales, to differences between individuals and experience over the life-course. It is unclear whether the changes from the species to the individual as being classifiable as *r* or *K*, and from the causally relevant variability and unpredictability being within a lifetime as opposed to over evolutionary time, should be viewed as a direct application of Pianka's theory (i.e. Pianka's theory *actually predicts* the same to hold for individuals as species, and lifetimes as epochal timescales), or an analogical extension (i.e. individual people are *analogous to* species in that they can be arrayed on a fast–slow continuum, and having an unpredictable childhood is *analogous to* evolving in an epoch where the climate changes a lot). The difficulty of resolving this question stems from the fact that Pianka's account of *r*/*K* differences was not based on a formal model that can be adjudged either applicable or not applicable to the scenario that LHT-P authors were using it for.

Authors in psychology did notice that the *r*/*K* framework had been largely abandoned in LHT-E. One consequence was the disappearance of the ‘differential K’ terminology and the rapid rise of the more neutral term ‘LHT’ in psychology (figure 1). The *r*/*K* descriptive distinction was retained but renamed the ‘fast–slow continuum’, to stay in step with later presentations of the principal-axis idea in LHT-E [46,54]. The key explanatory part of the *r/K* model (that ‘faster’ life histories were the result of variable or unpredictable environments) was also retained, though ‘variable or unpredictable’ tended to become ‘harsh or unpredictable’ [55]. With *r* and *K* selection now gone, the claim about environments was now justified with reference to the idea that higher extrinsic mortality rates select for earlier reproduction and greater reproductive effort. This claim, which dates back to Williams [56], is a widespread mid-level idea within LHT-E. However, more recent models show that truly extrinsic mortality does not change the age distribution of the population and thus has no direct effect on selection for any trait [57,58], although it can have indirect effects via increased population density and intensified selection on competitive ability [58]. Sources of mortality often described as ‘extrinsic’ may actually affect older individuals more strongly than younger ones, and this does indeed relax selection on late-life survival [57]. Mortality that differentially affects juveniles actually *strengthens* selection for late-life survival [57], which strikes directly against the LHT-P idea that a harsh childhood environment might be particularly important in ‘speeding up’ life-history strategy.

Another influential development within LHT-P was the argument that plasticity and genetic evolution might be equifinal. That is, if harsh or unpredictable regimes select for genetic variants that accelerate development, they should also select for plastic mechanisms that allow individuals to shift strategically towards accelerated development if they personally experience harshness or unpredictability. This move is what allowed the shift in focus, within LHT-P, to strategic responses to individual environmental variables (table 2). The move is intuitive: the parallel between tanning and genetic variation in skin colour provides a familiar exemplar of how plasticity does in the short term what selection does in the very long term. However, it is not theoretically trivial. Appropriate models (of which there are currently few) are required, and they predict circumstances under which plastic responses and effects of selection can be decoupled [19].

As more psychologists entered the arena of LHT, particularly since 2010, they added additional psychological traits that they were interested in to the ‘fast–slow’ umbrella. The criteria for doing this seem to be, partly, the existence of some intuitive reason why that particular trait could help individuals achieve more rapid reproduction (e.g. impulsivity or future discounting [26]); and partly whether, empirically, the trait does indeed correlate with other psychometric variables already deemed to be ‘fast’ (see for example [59] on obesity). This means, in effect, that an important part of the grounds for saying that LHT predicts two traits will correlate is that they do in fact correlate. This is clearly a different, more inductive type of theory-building to the explicit *a priori* modelling of LHT-E. This should not surprise us: LHT-P has perhaps adopted the mode of theorizing of psychology more broadly, whereas LHT-E has stuck with its particular mode of theorizing. Again, we emphasize that we document these differences without judgement: different disciplines theorize in different ways in part because their aims and subject matters are different. Understandably, given the typical disciplinary concerns of psychology, LHT-P has become largely an account of proximal psychological processes. This is exemplified in the quotation from [35, p. 1] reproduced in §2: ‘At its core…LHT is a motivational framework’. In other words, while LHT-E is predominantly a framework for generating formal models aiming to produce ultimate explanations, LHT-P is predominantly a framework for non-formal theorizing, describing empirical patterns and investigating proximate causes [20].

Many authors within LHT-P are aware that the moves from species differences to individual differences, from selection to plasticity, and from strict life-history traits to a broader suite of behavioural, motivational and attitudinal traits, constitute extensions of the LHT-E framework. They have written about how these moves can be justified [55,60,61]. Moreover, critiques of LHT-P's core claims have begun to be generated from within LHT-P itself [21,62,63]. It is not our purpose to evaluate those justifications or review those critiques here. We just wish to point out that neither the justifications nor the critiques feature formal modelling, as formal modelling is not a methodological rule of LHT-P. The justifications rely on plausible analogies (it is intuitive that the effect of personal environment on an individual ought to be the same as the effect of selective environments on populations, or that you should reproduce fast if you are likely to die sooner). The critiques often come down to empirical matters such as how much variation in individual psychological differences can or cannot be explained by a principal fast–slow axis [21,64,65] and what that means [62]. Thus, LHT-P and LHT-E are currently talking past one another.

## 5. Prospects for future integration of life-history theory

We have argued that LHT-E and LHT-P have developed as two largely separate research programmes, with different aims, different interests and different modes of theorizing. This state of affairs causes difficulties if the two are not clearly distinguished. For example, were the core assumptions of LHT-P to be refuted, readers might believe that LHT-E had fallen; or LHT-E might be invoked to support claims of LHT-P that actually have no formal evolutionary basis. Awareness of the distinction will be essential in building the emerging bridge between the areas of life history and learning. Broadly, this interface might have two foci: species-typical development and individual differences. The former focuses on how *species*-*typical* learning capacities relate to *species-typical* life-history variables; this area is relatively mature (as evidenced by the examples in this volume). The latter focuses on *individual differences* in learning patterns in relation to *individual differences* in life-history variables; this area is relatively new. In humans and rats, for instance, exposure to psychosocial adversity may shorten development, accelerating the onset of certain learning abilities (e.g. aversive fear conditioning, needed to navigate the world independently), while reducing others (e.g. attachment-related learning, forming a preference for cues associated with the parent) [66]. Exactly how evolutionary thinking should be deployed in understanding these phenomena is an area for future theory development.

We have argued that although LHT-E and LHT-P are clearly distinct, they are also historically and conceptually linked. Rather than abandoning those links, we believe researchers should strengthen them. Psychological theories do need to be grounded more deeply in our understanding of evolutionary processes and evolutionary history. We conclude with a few observations about how this strengthening might be done.

Formal evolutionary models should be developed to explore the concrete situations LHT-P is interested in. For example, although many LHT-E models deal explicitly with how multiple life-history traits should covary across species [67], fewer have explicitly dealt with how traits should covary across individuals within the same population [68]. This is challenging because it will depend on the genetic and developmental architecture of the traits, but predictive frameworks are being developed [51]. Modelling should be extended to incorporate behavioural and psychological traits as well as life-history traits [51]. For example, models have begun to appear predicting when individuals should be impulsive (in the sense of discounting the future heavily), depending on their environment and current state [69,70]. Importantly, these models do not rely on extrinsic mortality as the sole or even main explanatory factor, and they make potentially testable predictions. However, the models are complex: the predicted outcome depends on the precise assumptions about the structure of the environment and about the mapping between the behaviour and fitness. Thus, as well as developing the models and trying to test their predictions, it will be critical to gather data from natural human populations in order to validate, as far as possible, the modelling assumptions.

Formal modelling should also be applied to LHT-P's claims about developmental plasticity. The claims within LHT-P that childhood experience should be a key accelerator of life-history strategy are potentially problematic in a number of ways. Increased juvenile mortality, unless adult mortality is also increased, should if anything slow life history down (see §4). It is also, as noted above, problematic to assume that the effects of plasticity on the phenotype should necessarily evolve to look the same as the effects of selection. If the argument is that childhood experience serves as a ‘weather forecast’ of the conditions that will be experienced in future in adult life [71], the validity of this argument depends on assumptions about the statistical structure of environments [72], assumptions that needed to be validated empirically [73].

In short, there is scope for a programme of work establishing an ultimate evolutionary basis for the key claims of LHT-P: of a fast–slow continuum of individuals; of the inclusion within it of some psychological traits but presumably not others; and of evolved plasticity using childhood cues to calibrate the position on this axis. The appeal of this kind of work is that would allow psychologists to ground their claims in evolutionary theory in a way that evolutionary ecologists also feel satisfied by, as the grounding would have used the methodological rules of that discipline. We hope that more collaboration will develop between evolutionary modellers and empirical psychologists.

If LHT-P could move closer to LHT-E by adopting a more formal approach, evolutionary biology could also be more informed by LHT-P's concerns with proximate cognition, and with empirical patterns of individual differences. There has been an increased focus on individual differences in ecological research recently, and the covariance structure of those individual differences is an important concern [74]. Much of this research is organized within the ‘pace-of-life’ framework [75], whose findings speak directly to LHT-P. Key empirical findings are that the genetic correlations between traits are often different from the phenotypic correlations; and can also differ between populations of the same species [76]. This could provide important impetus for extending the measurement of a ‘fast–slow’ continuum beyond a narrow range of Western research participants: perhaps the key generalizations are restricted to certain physical and social environments (see also [77]). Thus, there is scope for a comparative empirical science of individual differences and trait covariation that could be initially data-focused and agnostic about the evolutionary or environmental factors responsible for the patterns.

In conclusion, although LHT-E and LHT-P currently have less in common than their names imply, they share historical sources. Importantly, they could come into much closer relationship in the future, by sharing both methodological resources and empirical generalizations across the divide. This would contribute to the integration of human psychology into the more general framework of organismal biology, and be to the scientific benefit of both sides.

## Data accessibility

This article has no additional data.
